# The Effect of Alternative vs. Focal Identity Accessibility on the Intent to Purchase Products: An Exploratory Study Based on Chinese Culture

**DOI:** 10.3389/fpsyg.2022.852505

**Published:** 2022-06-07

**Authors:** Fei Chen, Cheng Cheng Yan, Lin Wang, Xiao Jing Lou

**Affiliations:** ^1^College of Business Administration, Ningbo University of Finance and Economics (NBUFE), Ningbo, China; ^2^Faculty of Mechanical Engineering and Mechanics, Ningbo University, Ningbo, China; ^3^College of Finance and Information, Ningbo University of Finance and Economics (NBUFE), Ningbo, China

**Keywords:** focal and alternative identity, Chinese culture, identity accessibility, self-clarity, self-discrepancy

## Abstract

Much of early western research has focused on identity. A primed identity can inhibit the priming of other alternative identities, and also negatively affect the intention to purchase products related to those alternative identities. In western culture, individuals operate within a cultural framework that makes them more likely to prioritize their own goals and less likely to rely on environmental factors when evaluating others. Individuals are more likely to choose products that fit their primed identity. In this study, we suggest that in collectivist Chinese culture, individuals may give priority to group-level goals and attach more importance to situational factors when evaluating others. Therefore, a primed identity may not necessarily inhibit the priming of alternative identities. In this research, we examine the association between a focal identity and alternative identities, with particular emphasis on the accessibility, discrepancies, and effects on purchase intention of alternative identities. We also examine the intent to purchase products related to the alternative identity vs. the focal identity, developing a model with which to explore this construct. We test four hypotheses through experiments using an online questionnaire and analyzing the resulting data using statistical product service solutions (SPSS) 22.0 and the PROCESS macro modeling tool. The results are as follows: First, the association between a focal identity and alternative identity has a positive impact on the accessibility of the alternative identity. The clearer the alternative identity is, the greater the impact of the association between the focal identity and alternative identity on the latter’s accessibility. Second, the accessibility of the alternative identity has a positive impact on the intent to purchase alternative identity vs. focal identity-related products. The greater the discrepancy between the focal identity and the alternative identity, the greater the impact on the intent to purchase alternative identity vs. focal identity-related products. The results of this study confirmed the interaction between identity association and the clarity of the alternative identity, as well as the influence of the accessibility of the alternative identity on the intent to purchase alternative identity vs. focal identity-related products. We contribute to the development of a theory on intention to purchase identity-related products.

## Introduction

Individuals obtain new identities over time and in different situations (Ryan and Deci, 2012). Identities occur to and make sense depending on the subjects (e.g., other people, groups, or organizations) with which individuals interact. When individuals leave their original environment for another, they may adopt new identities that accord with the new environment. New identities may also be adopted because of curiosity or interests (Deci, 1992). Different identities allow individuals to adapt and respond to life’s multiple demands.

Individual behaviors are identity-driven ones. Individuals pay more attention to identity-relevant information and make decisions based on their focal identities. Studies have shown that multiple identities are organized within a network of associations, and that these identities may vary according to their degree of association, ranging from complete separation to complete association. The effect of identity activation on behavioral response is so powerful that even if the primed individual has an alternate identity opposite to the focal identity, priming-consistent behavior is often observed (Chen and Bond, 2010; Coleman and Williams, 2013). That is, the activation of one identity inhibits the activation of another one. Previous studies have examined the effects of conflicting alternative identities on the choice decision and post-decision satisfaction (LeBoeuf et al., 2010), and the effect of balancing difficulty on focal and alternative identities (Saint Clair and Forehand, 2020).

Culture may be considered as a framework which encompasses people of similar faith, values, and meaning, or the sum total of material and spiritual productive capacity acquired in the course of social practice and creation of material and spiritual wealth (Saint Clair and Forehand, 2020). Cultural values are the common standards and dominant patterns that a particular group (e.g., a society) may have at a given time (Saint Clair and Forehand, 2020). In individualistic western culture, individuals operate within a cultural framework that makes them more likely to prioritize their own goals and less likely to rely on environmental factors when evaluating others (Saint Clair and Forehand, 2020). This confirms the results of the western research that we mentioned above. These studies have drawn the following conclusions: The activation of an identity inhibits the activation of another identity, and priming consistent behavior toward the primed identity can be seen as a consequence of the individual’s the cultural framework (Abrams and Hogg, 1988; Coleman and Williams, 2013). However, in collectivist Chinese culture, individuals may prioritize group-level goals and attach more importance to situational factors when evaluating others (Saint Clair and Forehand, 2020). How individuals under the collectivist Chinese cultural framework will behave is still unknown.

Previous researchers have proved the effect of self-concept clarity on non-conformity of self-definition (Rios Morrison and Wheeler, 2010), self-esteem (Rahimi and Strube, 2007), body image (Vartanian, 2009), and psychological adjustment (Bigler et al., 2001). Many have also demonstrated the moderating effect of self-concept clarity on women’s triggering themselves objectively in the context of materialism (Teng et al., 2016). A high level of self-concept clarity allows individuals to experience certainty and clarity about their identity. In contrast, when individuals have a low level of self-concept clarity, they may not be exactly sure of who they are. Clarity of identity thus seems to play an important role in behavior and choice tendency.

Researchers have also paid considerable attention to the emotional consequences caused by self-discrepancy (Higgins, 1987), while ignoring the motivational effect that is caused by it. In this study, we demonstrate that when individuals perceive a deficiency in their identity, they take steps to reduce or eliminate that deficiency.

The purposes of this study are as follows: First, we investigate the effects of the association between focal identity and alternative identity on the accessibility of alternative identity in the following two different settings: Individualistic western culture and collectivist Chinese culture. Second, we explore the effect of accessibility of alternative identity on intention to purchase alternative identity vs. focal identity-related products. Third, we examine the moderating role of self-concept clarity in the relationship between focal identity and accessibility of the alternative identity, postulating that self-concept clarity and self-discrepancy play moderating roles in the relationship between the accessibility of alternative identity and its effect on intention to purchase products related to the alternative vs. focal identity.

## Identity Hierarchy

Shah et al. (2003) proposed a goal system framework to clarify the hierarchy between goals and means and account for how goals are pursued. Goals are considered as a kind of knowledge structure guided by cognition in this framework. According to the authors, correlation is a critical cognitive factor in any goal system on the lateral level. More specifically, a goal can be connected with another goal in the same situation, and means may be connected with other means that can both satisfy a given goal. A means can serve to satisfy several goals at the same time due to multi-finality. The degree to which a means is associated with a goal is inversely proportional to the number of goals associated with the means (Förster et al., 2007). In addition, between the goals and the means on the lateral level, there is a negative correlation. According to Shah et al. (2002), the appearance and activation of a goal inevitably involves the shielding of other goals (i.e., enhanced competitive relationship). A positive correlation also arises on the vertical level between goals and sub-goals. Empirical studies prove that resolving a focal goal into sub-goals makes it easier to pursue the focal goal, increasing both motivation and persistence (Locke and Latham, 1990; Brunstein, 1993; Soman and Shi, 2003). Sub-goals and motivation increase the likelihood of success (Huang et al., 2017).

In addition to the cognitive principles, the goal system has certain motivational characteristics (Shah et al., 2003). As empirical studies have proven, people have multiple needs at any given time. Those needs transform into motivation and drive people to act as long as they are sufficiently aroused (Kotler et al., 2018). Thus, motivation can be described as an internal incentive that drives people to achieve goals (Park, 1985). Hoyer and MacInnis (2008) highlighted that the consequence of motivation is to drive people to make strong efforts to achieve their goals. When people are positively motivated to achieve goals, they are willing to think about them, seek relevant information, and try their best to assess and remember information related to them. Thus, motivation affects how people deal with information and make decisions to achieve goals (MacKenzie and Spreng, 1992).

In brief, goal system theory gives insight into how goals interact with other goals and with the means (e.g., substitution) to satisfy them (Shah et al., 2003). It reveals how people solve the problems involved in pursuing a goal and selecting the means to achieve them. In this study, we introduce the identity structure.

Empirical researchers have defined identity in different ways. For example, social psychologists have proposed personal identity and social identity (Stryker and Burke, 2000). Personal identity refers to how individuals feel about themselves, and social identity refers to individuals as members of a group and their feelings about the group and its members.

Higgins (1987) pointed out the following two factors needed to clarify the self: Domains of the self and the standpoint. The first includes the following three kinds of the self: The “actual self” refers to an individual’s actual attributes; the “ought self” refers to the attributes that one feels one ought to have (e.g., duty and responsibilities); and the “ideal self” refers to attributes that one wants to have (e.g., hopes and wishes).

According to Higgins (1987), six kinds of self-state can be identified by combining the self-domains and points of view. Actual/own and actual/others correspond to the two self-concepts mentioned earlier (Oyserman, 2004), while ideal/own, ideal/other, ought/own, and ought/other are four self-guide standards (Higgins et al., 1986). However, not all people have both self-guides (i.e., ought self-guides and ideal self-guides). For example, a working mother who wants to be successful in her job has an ideal self-guide rather than an ought self-guide, whereas one who thinks she is responsible for being a good mother may have a strong ought self-guide. People are more likely to make comparisons between their behaviors and contextual standards when they pay attention to the self. The motivation to do so arises from a perceived discrepancy, which drives behavior congruent with those standards (Duval and Wicklund, 1972). In this study, the pursuit of identity (either actual or ideal) can be considered as the pursuit of a goal, and the identity hierarchy has the same function as a goal hierarchy.

## The Association Between Focal and Alternative Identities and Its Effect on Accessibility of the Alternative Identity

Examining the association between identities, one point of view shows that people structure their identities using association networks; those identities are predicted as ranging from total association to total disassociation (Amiot et al., 2007; Luna et al., 2008). Identities vary based on different identity-based needs, such as cognition and behavior, among which people try their best to maintain harmony (Lane and Scott, 2007; McConnell, 2011). The idea of an association of multiple identities was also put forward by Roccas and Brewer (2002). In their opinion, identities are perceived as merged or integrated, which is synonymous with what we refer to in this study as associated. Identities that are perceived to be distinct and separated are referred to as disassociated (Saint Clair and Forehand, 2020). Several empirical studies consider associated and disassociated identities as two totally different structures (described earlier as merged or integrated, distinct or separated). We herein claim that those two distinct structures can be seen as one continuum, in which the most associated and disassociated identities represent the endpoints.

A previous research has demonstrated that association between identities has different effects on those identities (Hugenberg and Bodenhausen, 2004). More specifically, when two identities are less associated (i.e., dissociated), the primed identity is more likely to inhibit the priming of another identity. That is, people tend to avoid the alternate identity when the focal and alternate identities are disassociated (Zhang and Khare, 2009).

The extent to which identities are associated depends on several cues. First, coactivation of several identities has a positive effect on identity association (van Osselaer and Janiszewski, 2001; Sternberg and McClelland, 2012). Stets (1995) explored the relationship between role identity and personal identity and demonstrated that this relationship exists because these two types of identity share a common meaning concerning the extent to which people control all aspects of their environment. Identities are also selectively activated to fit the meaning depending on the environment (Heise, 1979). Thus, those identities with a common meaning are predicted to be associated simultaneously as long as meaning arises in the environment; for example, leadership, effort, or improvement can be considered as characteristics of both household head identity and worker identity. Second, the degree to which identities can replace each other can have a positive effect on their association (Saint Clair and Forehand, 2020). For example, people with jobs in infant education or kindergarten can also take care of their own children as a worker as well as a parent; in this situation, they may treat those two identities as substitutable with each other. Third, overlapping identities may prompt identity association (Roccas and Brewer, 2002; Amiot et al., 2007). As previously mentioned, each identity has its unique content. Identities may overlap to different degrees because of their content (e.g., self-complexity). For example, for someone who graduated with a master’s degree and works as a teacher in a high school, the graduate school identity and the teacher identity are highly overlapped, because both identities are connected with school and acquisition of knowledge. From another viewpoint, overlap of these two identities may be perceived as low when considering the differences between these identities in terms of financial content (i.e., tuition and salary). Thus, identity overlap refers to nodes of information stored in memory that are perceived to be connected with other nodes. A high number of connected nodes leads to a high overlap of identities, thereby leading to a high association between those identities. According to the theory of spreading activation, which states that when people perceive a common node, they automatically think of other nodes, in this study, we hypothesize the following:

H1: The association between focal and alternative identities will positively affect the accessibility of alternative identity.

## The Effect of Accessibility of Alternative Identity on Intent to Purchase Identity-Related Products

Identity accessibility occurs when people hold a positive attitude toward an identity (Wheeler et al., 2005). When an identity is accessible, people are more likely to act on the stimuli related to that identity (Zhang and Khare, 2009). Taking into account the proactive interference effect, in which information acquired early impedes the memory of information acquired later (Hoyer and MacInnis, 2008), we postulate that a consumer decides to buy a particular product depending in part on the focal goal that the product serves (van Osselaer and Janiszewski, 2012). In contrast, with the retroactive interference effect, information acquired later impedes the memory of information obtained earlier. Accessing alternative identity could help individuals achieve additional identity-related goals rather than not accessing the alternative identity. Accessing the alternative identity thus leads to perceiving the focal identity as of relatively lower importance, which also reduces intent to buy products related to the focal identity.

Comparisons are often made between alternative identities when making purchasing decisions. Past research has not given much attention to the roles of alternative identity in making purchase decisions and choosing products with consideration of opportunity costs. Recent work has explored other contexts, including replacement choices when no options are available in a display set (Karmarkar, 2017), and avoiding choice (for example, avoiding an unhealthy snack for a dieter) (Arens and Hamilton, 2016).

Certain products are likely to appeal to consumers’ focal identity (e.g., work clothes for working men or women). In contrast, other products are likely to serve the alternative identity accessed from memory (e.g., baby clothes for a mother or father). Since consumers can have multiple goals (Fishbach and Dhar, 2008), the importance of any single goal may differ according to the degree to which other goals are pursued and other identities also activated at the same time. When an identity is activated, it might become more accessible in memory, and consumers might evaluate its associated means more positively (van Osselaer and Janiszewski, 2012). The intent to purchase products related to the focal identity may be lower after accessing an alternative identity than after only considering the focal identity. Therefore, we hypothesize that:

H2: The accessibility of alternative identity will positively affect the intent to purchase alternative identity vs. focal identity-related products.

## The Moderating Role of Self-Concept Clarity

Self-concept clarity is the extent to which the contents of one’s self-concept (e.g., the attributes that are perceived by an individual) are clearly and confidently defined, organized, internally consistent, and temporally stable (Campbell et al., 1996). Self-concept clarity, which is essential to daily life, represents the extent of self-belief, not the right or wrong of self-belief. The previous researchers found that lack of clarity of self-concept can be an indicator of low functioning, depression, and anxiety (Bigler et al., 2001; Campbell et al., 2003). It also reduces positive feedback about failure (Stucke and Sporer, 2002). According to Campbell et al. (1996), self-concept and self-esteem are positively correlated, whereas self-concept clarity is negatively correlated with anxiety, depression, and neuroticism.

The notion of self-concept clarity is independent from the contents of self-concept (e.g., perceived attributes), which can encompass varying degrees of confidence and stability. For example, some researchers hold the opinion that self-concept clarity is a concomitant of self-esteem. Therefore, people with high self-esteem will always have positive, clear beliefs about the self, but those with low self-esteem may not have negative, clear beliefs about the self. Instead, their descriptions about the self may be neutral, inconsistent, and unstable; thus, they have low self-concept clarity (Campbell and Lavallee, 1993).

Self-concept clarity can vary in amount, as self-concept is a limited resource that individuals must make very careful use of Shah et al. (2012). The different levels of self-concept clarity may elicit different responses to external stimuli; for example, individuals with low self-concept clarity are more likely to perceive and be influenced by external stimuli. Vartanian (2009) confirmed the inverse relationship between self-concept clarity and the internalization of cultural ideals of attractiveness among women. Emery et al. (2015) examined the relationship between self-concept clarity and self-expansion. In that experiment, people with high self-concept clarity were more likely to self-expand than others, while those with low self-concept clarity had less interest in self-expanding (e.g., showing even less interest in romantic relationships).

In this study, self-concept clarity refers to perceived clarity and consistency about the self-concept (e.g., identity). It is the extent to which people clearly and confidently perceive their own attributes (Campbell et al., 1996). It is an uncontroverted fact that people tend to choose products or brands based on identity (Reed et al., 2012). People may be clearly confident about whom they should be, what they should believe, and what they should do when they have high self-concept clarity related to the alternative identity. Therefore, we hypothesize the following:

H3: Self-concept clarity related to the alternative identity plays a moderating role in the effect of the association between focal and alternative identities on the accessibility of alternative identity.

## Self-Discrepancy and Its Moderating Role

Self-discrepancy depends on the self-guides (e.g., ought self and ideal self) that people are motivated to meet (Higgins, 1987) and the extent to which they are motivated to decrease differences. Empirical studies have demonstrated the significance of motivation. For example, James (1984) insisted that these guides have a positive effect in promoting behavior that can reduce differences and evoke emotions (e.g., disappointment and dissatisfaction). According to aspiration theory, people must have those “ideal” goals to perform better (Lewin, 1936). In addition, according to control theory, self-discrepancy is considered as negative feedback, self-concept is seen as sensed value, and self-guides are seen as the standard (Wiener, 1948).

The previous research has revealed a series of emotional consequences caused by self-discrepancy. For example, individuals with high self-discrepancy are more likely to experience emotional distress like disappointment and dissatisfaction (Strauman and Higgins, 1988) and low self-esteem (Moretti and Tory Higgins, 1990). In addition, Bessenoff (2006) revealed that self-discrepancy may moderate the tendency to make comparisons with the external. For instance, women with body self-discrepancy are more likely to relate failure of achieving the ideal body to their self-concept (Bessenoff, 2004). Nevertheless, comparison is not necessarily associated with self-discrepancy. Not only women who are unsatisfied with their body image make comparisons; those who are less dissatisfied with their body image may have already had an ideal body shape, or their self-worth may have nothing to do with their body shape. That is, low dissatisfaction may not cause the perception of self-discrepancy.

Individuals with high self-discrepancy may perceive differences between the actual self and ideal self (e.g., the ought self), and may therefore be motivated to decrease differences. For example, a woman who considers herself an inadequate mother (i.e., the absence of mother identity and obligation of the ought self) may be motivated to take steps to fulfill the perceived obligation by paying more attention to her child (e.g., cooking and buying toys). For people with low self-discrepancy, such differences may not be perceived and therefore no motivation may arise. Even those with a strong sense of self may do nothing because they may not perceive the differences between the actual self and self-guides (e.g., ought self and ideal self).

In the extant literature, self-discrepancy theory proposes the following consequential mechanism: that if the deficit between the actual and the ought self increases, the deficit may have a negative effect on the actual self. Under such circumstances, individuals may be motivated to eliminate the deficit. Thus, we hypothesize that the following:

H4: Alternative identity self-discrepancy will play a moderating role in the effect of accessibility of alternative identity on the intent to purchase alternative identity vs. focal identity-related products.

**FIGURE 1:**
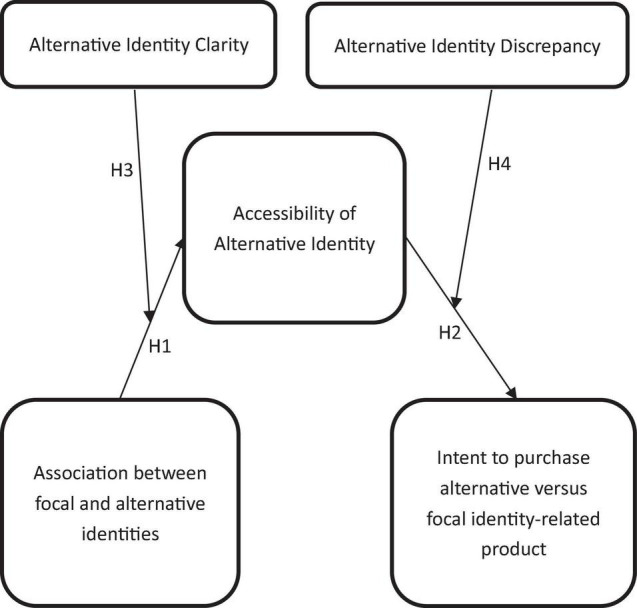
Research model.

In summary, all hypotheses in this study are shown in Figure 1.

## Research Overview

### Identity Setting and Objects Selection

Work seems to be an essential part for most of us. We can maintain daily lives and pay for it through working. In addition, work is related to other aspects of our lives, we choose what to do, and what we do will identify what we are (Gini, 1998). Similarly, household head is closely related to all of us. This study takes the household head identity as the focal (ideal) identity and the worker identity as alternative (ought) identity, because most people consider themselves are commonly related to those two identities (Angle and Forehand, 2016). Thus, we choose working parents as empirical objects in this study.

### Scenario Development

According to the worker identity (focal identity) and the household head identity (alternative identity) we chose, we developed two different scenarios as follows: Scenario 1 described some attributes of people as a household head. Scenario 2 described some attributes of people as a working man or woman. The statements were developed as follow:

Scenario 1: As a head of household only with family members, I like my household work and I feel obligation to my family; therefore, I am willing to wake up early to spend more time to the household work in the morning before going to my company. When I feel difficulties in the process of doing the work for my family, I would try to find the interests of each family member actively. It is my duty to provide a good living environment to my family, which will help it avoid from illness and external harm. Besides, making more time to the children is my responsibility to improve their education level. The works are required to maintain the health of me and my family.

Scenario 2: As a worker of a company, I like my job very much and I am eager to succeed. Therefore, I am willing to come to the company early every day to spend more time on work. When I have difficulties, I would like to solve problems with other colleagues actively. I feel fulfilled and everything I do is of value to myself. I think I can move up to this job and hopefully get the success I want. My families have great wishes for me because they think that I find the work I really like. The work will improve the life of me and my family.

## Main Study

### Manipulation of Priming the Focal Identity

After reading Scenario 1, the participants were asked to complete questionnaire, this study processed the focal identity priming by adapting the sentence-completion task (Srull and Wyer, 1980); the participants were asked to make meaningful sentences with scrambled words, and all scrambled words in each content from the Scenario 1 including “spend time, for, duty, my, children, to, me, it, is,” “I, responsible, think, for, family, my, I’m,” “obligation, my, to, for, family, it’s, maintain, health,” “like, household, I, work,” and “do, is, responsible, everything, I, for, family, my.” Then, the participants were required to indicate the extent to which they felt each of attribute of the scenario. The questionnaire was composed with three items including “I feel obligation,” “I feel duty,” and “I feel responsibility.” All items were measured by a 7-point scales (1 = not at all, 7 = very much).

Then, the participants were exposed to Scenario 2. The participants were asked to complete another questionnaire to indicate the extent to which they felt each of attribute of the scenario after reading Scenario 2, the questionnaire was composed with three items including “I feel obligation,” “I feel duty,” and “I feel responsibility.” All items were measured by a 7-point scales (1 = not at all, 7 = very much). Then, the participants were asked to indicate by which points (effect, good environment, improvement, leadership, others) they felt similarity or dissimilarity between Scenario 1 and Scenario 2.

### Measurement of Association Between Focal and Alternative Identity

As we mentioned in identity setting part, this study took the household head identity as the focal identity, and the worker identity as the ought identity. To test the association between identities, the participants were asked to complete the identities association questionnaire with the condition that “How do you think of the relationship of the work identity and the household head identity described in scenarios?” “Please mark “√” to the number showing your thought or opinion,” the measure of association include four items as follows: “In general, I think the work identity is similar with the household head identity,” “In general, I think the work identity and the household head identity do overlap,” “In general, I think the work identity is related to the household head identity,” and “In general, I think the work identity is associated with the household head identity” (Saint Clair and Forehand, 2020). All items were measured by a 7-point scales (1 = not at all, 7 = very much).

### Measurement of Alternative Identity Clarity

The participants were required to complete identity clarity-relevant questionnaire with the condition that “Please mark “√” to the number showing your thought or opinion.” Three items were used to test the alternative identity clarity. “If I was asked to describe me as a worker, my description might end up being same from 1 day to another,” “My beliefs about the worker identity-relevant events seem to be relatively stable,” “I would tell someone what kind of worker I really ought to be if needed” (Zhang and Khare, 2009). All items were measured by a 7-point scales (1 = not at all, 7 = very much).

### Measurement of the Accessibility of the Alternative Identity

The participants were required to complete another questionnaire that measure the accessibility of the alternative identity. This questionnaire including four items: “There have often been the cases of my thinking I am a worker,” “The thoughts about worker identity are vivid in my memory,” “The worker identity is easily accessed from my memory,” “There is the worker identity concept in my memory” (Zhang and Khare, 2009). All items were measured by a 7-point scales (1 = not at all, 7 = very much).

### Measurement of the Alternative Identity Discrepancy

The participants were required to complete identity discrepancy-relevant questionnaire with the condition that “Read the attributes that you as a head of household should or ought to possess carefully, and rate the extent to which you feel the deficits in view of each of attributes listed below.” To test the alternative identity clarity, four items were adapted and modified from Van Hook and Higgins (1988) research. “I feel the deficits in view of obligation to the work and company,” “I feel the deficits in view of solving problems with other colleagues actively,” “I feel the deficits in view of moving up to this job and hopefully getting the success I want,” and “I feel the deficits in view of spending time on work.” All items were measured by a 7-point scales (1 = not at all, 7 = very much).

### Measurement of the Intent to Purchase Alternative vs. Focal Identity-Related Product

In this part, the participants were first exposed to statements of product A and product B; product A was defined as “cloth product A is for your son or daughter, the price of this product is 650 RMB; whereas, product B is for you as a working man or woman, the price of this product is 650 RMB.”

After reading the information for product A and B, the participants were asked to finish three purchase intent tasks. First, they were asked to complete a questionnaire with the condition that “Imaging your visiting to the department store for buying clothes, please mark “√” to the number showing your thought or opinion about making purchase decision,” the questionnaire was composed with the following four items: “I would like to buy,” “I have more intent to buy,” “There is high possibility that I buy,” and “I feel more attraction to buy.” All items were measured by a 7-point scales (1 = product A for children, 7 = product B for me). Second, the participants were asked to indicate their purchase intent to product A by the following four items: “I would like to buy product for my son or daughter rather than for me,” “I have more attention to purchase product for my son or daughter rather than for me.” “There is high possibility that I buy product for my son or daughter rather than for me,” and “I feel more attraction to purchasing product for my son or daughter rather than for me.” Third, the participants were also asked to indicate their purchase intent to product B by same items with product A. “I would like to buy product for me rather than for my son or daughter,” “I have more attention to purchase product for me rather than for my son or daughter,” “There is high possibility that I buy product for me rather than for my son or daughter,” and “I feel more attraction to purchasing product for me rather than for my son or daughter.” All items were measured by a 7-point scales (1 = not at all, 7 = very much).

Lastly, the participants were asked about their basic demographic information including gender, age, incomes and nationality. Besides, the participants’ information about whether they have son or daughter or not, and their job category were also asked.

### Data Collection

First, we finished questionnaire in English according to the measurement we mentioned before, then, we translated the questionnaire into Chinese and asked a professional company to complete the questionnaires, and promised 6 RMB reward for each of the participants. The participants who participated in the current questionnaire were asked to meet the following requirements: First, those who had the worker identity and the household head identity. Second, those who had at least one child could participate in the questionnaire. Last, only those who lived with their families could participate in the questionnaire.

A total number of respondents for the questionnaires were 300, and 284 of them were used in this study for the hypotheses test, and 16 of them were excluded because they did not live with their family.

## Analysis and Results

### Demographic Analysis Results

As shown in Table 1, among the total 300 questionnaires, 284 were used in total, 31.3% (*N* = 89) of them were male, and 68.7% (*N* = 195) were female. The majority of those responders were aged between 21–30 years (48.2%) and 31–40 years (34.2%). All of them (*N* = 284, 100%) had either a son or a daughter. As for jobs, 41.1% (*N* = 117) of them worked in companies, 22.5% (*N* = 64) of them worked as public officers, 22.2% (*N* = 63) of them worked as freelancers, and 14.1% of them worked in other fields. That is, all these 288 participants met the requirements of the need for the identity we designed. As for the household incomes, 7.7% (*N* = 22) responders reported their household income was between 0 and 3,000 RMB; 40.1% (*N* = 114) of them were between 3,000 and 6,000 RMB, 24.6% (*N* = 70) of them were between 6,000 and 10,000 RMB, and 27.5% (*N* = 78) of them were more than 10,000 RMB.

**TABLE 1:** Demographic analysis result.

Variable		Frequency	Percentage (%)
Gender	Male	89	31.3
	Female	195	68.7
Age (years)	0–20	22	7.7
	21–30	137	48.2
	31–40	97	34.2
	41–50	23	8.1
	Over the age of 50	5	1.8
Child	Yes	284	100
	No	0	0
Job	A worker in company	117	41.2
	Public officer	64	22.5
	Freelancer	63	22.2
	Others	40	14.1
	No job	0	0
Income	0–3,000 RMB	22	7.7
	3,000–6,000 RMB	114	40.1
	6,000–1,000 RMB	70	24.6
	Over 10,000 RMB	78	27.5
Total response		284	100

### Reliability and Validity

To test the reliability and validity of the questionnaires, the statistical product service solutions (SPSS) 22.0 was used to analysis the five components. Results were shown in Table 2, five principal components’ total variances were 89.6%, *p* = 0.000 < 0.001, which meant a stronger correlation between those variables, and all those components were suitable for factor analysis. Besides, all of the Cronbach’s-α results > 0.7, which represented a good inconsistency of each construct.

**TABLE 2:** The reliability and validity results of the principal component.

		Component						
Construct	Item	1	2	3		4	5	Cronbach’s-α
Intent to purchase identity-related product (IP)	IP3	0.883	0.178	0.231		0.123	0.165	0.966
	IP2	0.879	0.159	0.180		0.167	0.224	
	IP4	0.875	0.218	0.180		0.138	0.250	
	IP1	0.863	0.163	0.288		0.122	0.168	
Alternative identity discrepancy (AID)	AID3	0.137	0.817	0.074		0.293	0.084	0.888
	AID2	0.095	0.812	0.177		0.281	−0.039	
	AID4	0.237	0.797	−0.011		0.202	0.018	
	AID1	0.183	0.770	0.201		0.283	−0.123	
Accessibility of alternative identity (AC)	AC1	0.234	0.071	0.820		0.130	0.230	0.898
	AC3	0.168	0.223	0.762		0.211	0.310	
	AC2	0.265	0.251	0.760		0.199	0.259	
	AC4	0.215	0.001	0.759		0.103	0.254	
Association between focal and alternative identity (AS)	AS2	0.150	0.283	0.141		0.821	0.099	0.894
	AS1	0.288	0.248	0.084		0.812	0.038	
	AS3	0.035	0.265	0.264		0.783	0.033	
	AS4	0.071	0.294	0.100		0.771	0.209	
Alternative identity clarity (AIC)	AIC3	0.254	−0.036	0.295		0.093	0.852	0.923
	AIC1	0.193	−0.024	0.337		0.125	0.834	
	AIC2	0.261	−0.009	0.287		0.097	0.830	
Eigen value			3.641		3.051		3.044	0.533
Variance explained			30.345		25.424		25.366	4.440
Variance cumulative			30.345		55.770		81.136	85.576
KOM measure of sampling adequacy							0.896	
Bartlett’s test of sphericity			Approximate Chi-square				4929.386	
			*df*				0.171	
			Sig.				0.000	

### Manipulation Checks

To measure the participants’ perception about identity attributes in each scenario, we used the One-Sample *T*-test in SPSS 22.0 to verify whether there was a significant difference between the average perceived value of attributes and 4 in the both scenarios. The results were as shown in Table 3. Besides, the Cronbach’s-α of the focal identity was 0.883, and the Cronbach’s-α of the alternative identity was 0.784. both of them were higher than 0.7, which represented a good inconsistency of each construct.

**TABLE 3:** Results of participants’ perception about focal and alternative identity in each scenario.

	Test value = 4							
	*N*	Mean	*t*	*df*	Sig.	Mean difference	95%	
							Lower	Upper
Focal identity	284	5.6033	27.154	283	0.000	1.60	1.4871	1.7195
Alternative identity	284	5.5915	26.610	283	0.000	1.59	1.4738	1.7093

As shown in Table 3, the participants’ perception about the focal and alternative identity were significant (Mean_*F*_ = 5.6033, *t* = 27.154, *p* < 0.001), (Mean_*A*_ = 5.5915, *t* = 26.610, *p* < 0.001). That is, the participants’ perceptions about both focal and alternative identities were significant.

**FIGURE 2:**
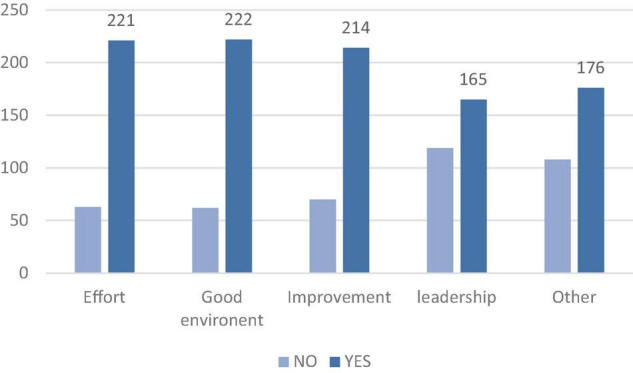
Results of participants’ thoughts about similarity or dissimilarity.

Then, we used frequencies in SPSS 22.0 to verify the participants’ perceptions of similarity or dissimilarity between the focal and the alternative identities. The results were shown as Figure 2. Most participants thought that the focal and alternative identities were similar in five attributes. That is, the identities that included in the scenarios were considered as associated.

### Testing Hypotheses

As mentioned earlier, the purposes of this study were to explore the effect of the association between focal and alternative identities on the accessibility of alternative identity, the effect of accessibility of alternative identity on the intent to purchase alternative vs. focal identity-related product, as well as the moderating effect of alternative identity clarity and alternative identity discrepancy. According to the PROCESS macro model discussed by Hayes (2017), this study used the Model 7 and Model 14 in PROCESS macro model to verify the hypotheses of this study.

**FIGURE 3:**
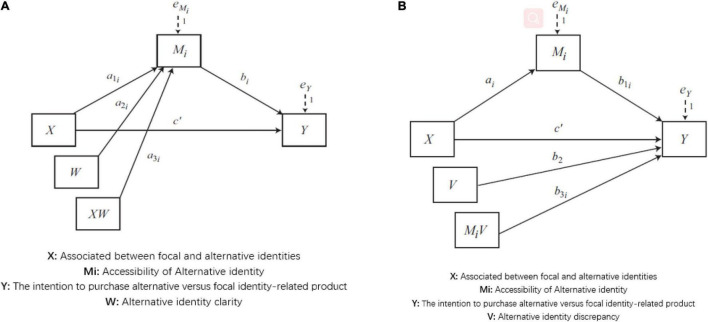
**(A)** Alternative identity clarity-related statistical diagram. **(B)** Alternative identity discrepancy-related statistical diagram.

The regression analysis analyzed the hypotheses of this study with two models. We used Model 7 in PROCESS macro model to verify the moderation effect of alternative identity clarity. As shown in Figure 3A, the first regression analysis mainly measures the effect of the independent variables on the dependent variable (accessibility of alternative identity), and the effect of accessibility of alternative identity on the intent to purchase alternative vs. focal identity-related product. Also, we used Model 14 in PROCESS macro model to verify the moderation effect of alternative identity discrepancy. As shown in Figure 3B, the second regression analysis measured the effect of the independent variables (accessibility of alternative identity, alternative identity discrepancy) on the intent to purchase alternative vs. focal identity-related product, and the effect of association between focal and alternative identities on the intent to purchase alternative vs. focal identity-related product. Besides, the independent variables and the moderator variables were likely to be highly correlated with their reaction, which might lead to the estimation problems caused by multi-collinearity and result in poor estimates of regression coefficients, large standard errors, and reduced power of the statistical test of the reaction; thus, all measured variables were mean-centered by the PROCESS macro model. Finally, the bootstraps of two regression analyses were designed as 5,000 times. The analysis results under the moderation effect of alternative identity clarity were listed as in Table 4A, and the analysis results under the moderation effect of alternative identity discrepancy were listed as in Table 4B.

**TABLE 4A:** Results of analyzing variables related to alternative identity accessibility.

Outcome: The accessibility of alternative identity							
	**Model summary**						
	***R***	***R* ^2^**	**MSE**	***F***	***df*1**	***df*2**	***p***

	0.6498	0.4222	0.8794	68.1973	3.0000	280.0000	0.0000

	**Model**						
	**Coefficient**	**Se**	***t***	***p***	**LLCI**	**ULCI**	

Constant	4.9929	0.0604	82.6069	0.0000	4.8739	5.1118	
AS	0.2946	0.0419	7.0369	0.0000	0.2122	0.3771	
AIC	0.3895	0.0537	7.2468	0.0000	0.2837	0.4953	
AS*AIC	0.2325	0.0318	7.3056	0.0000	0.1699	0.2952	

**Outcome: The intent to purchase alternative vs. focal identity-related product**							

	**Model summary**							
	***R***	***R* ^2^**	**MSE**	***F***	***df*1**	***df*2**	***p***

	0.4281	0.1833	1.6817	31.5316	2.0000	281.0000	0.0000

	**Model**						
	**Coefficient**	**Se**	***t***	***p***	**LLCI**	**ULCI**	

Constant	4.0487	0.3808	10.63333	0.0000	3.2992	4.7982	
AS	0.3229	0.0601	5.3748	0.0000	0.2046	0.4411	
AC	0.1760	0.0722	2.4378	0.0154	0.0339	0.3181	

**TABLE 4B:** Results of analyzing variables related to the intent to purchase alternative vs. focal identity-related product.

Outcome: The accessibility of alternative identity							
	**Model summary**						
	***R***	***R* ^2^**	**MSE**	***F***	***df*1**	***df*2**	***p***

	0.4928	0.2429	1.1442	90.4659	1	282.0000	0.0000

	**Model**						
	**Coefficient**	**Se**	***t***	***p***	**LLCI**	**ULCI**	

Constant	−2.055	0.2252	−9.125	0.0000	−2.498	–1.612	
AS	0.4101	0.0431	9.511	0.0000	0.3252	0.4950	

**Outcome: The intent to purchase alternative** vs. **focal identity-related product**							

	**Model summary**						
	***R***	***R* ^2^**	**MSE**	***F***	***df*1**	***df*2**	***p***

	0.7532	0.5673	0.8973	91.4648	4.0000	279.0000	0.0000

	**Model**						
	**Coefficient**	**Se**	***t***	***p***	**LLCI**	**ULCI**	

Constant	2.8267	0.2633	10.7340	0.0000	2.3084	3.3451	
AS	0.3533	0.0503	7.0207	0.0000	0.2543	0.4524	
AC	0.1547	0.0577	2.6798	0.0078	0.0411	0.2683	
AID	0.3889	0.0654	5.9442	0.0000	0.2601	0.5177	
AC*AID	0.4248	0.0280	15.1723	0.0000	0.3697	0.4799	

According to the results shown in Table 4A, *R*^2^ = 0.4222, *F*(3,280) = 68.2, *p* = 0.0000 < 0.001 indicated a good description of the alternative identity accessibility. To support our Hypothesis 1, the association between focal and alternative identities would positively affect the accessibility of the alternative identity (*b* = 0.2946, *t* = 7.0369, *p* = 0.0000 < 0.001). Besides, the accessibility of alternative identity is verified to have a positive effect on the intent to purchase alternative vs. focal identity-related product (*b* = 0.1760, *t* = 2.4378, *p* = 0.0154 < 0.05) under the alternative identity clarity’s moderation effect; thus, Hypothesis 2 was accepted.

Hypothesis 3 was also accepted. The alternative identity clarity played a moderating role in the effect of association on the accessibility of the alternative identity (*b* = 0.2325, *t* = 7.3056, *p* = 0.0000 < 0.001). More specifically, as shown in Table 5A, the conditional effects for the alternative identity clarity was significant at high (+1 SD) and mean levels, but not low (–1 SD) level of the alternative identity clarity [High_*Clarity*_ = 0.5682, 95%, CI (0.4597, 0.6767), Mean_*Clarity*_ = 0.2946, 95%, CI (0.2122, 0.3771), Low_*Clarity*_ = 0.211, 95%, CI (–0.0916, 0.1337)]; this finding suggested that when the alternative identity increased, the effect of association on the accessibility of alternative identity would also increase.

**TABLE 5A:** Results of testing the alternative identity clarity’s moderating role.

Moderator: Alternative identity clarity						
**Moderator level**	**Effect**	**Se**	***t***	***p***	**LLCI**	**ULCI**

−1.1766 (−1 SD)	0.211	0.0572	0.3681	0.7131	−0.0914	0.1337
0.000	0.2946	0.0419	7.0369	0.0000	0.2122	0.3771
1.1766 (+1 SD)	0.5682	0.0551	10.3118	0.0000	0.4597	0.6767

According to the results shown in Table 4B, *R*^2^ = 0.2429, *F*(1,282) = 90.5, *p* = 0.0000 < 0.001 indicated a good description of the alternative identity accessibility, and *R*^2^ = 0.5673, *F*(4,279) = 91.5, *p* = 0.0000 < 0.001 indicated a good description of the intent to purchase alternative vs. focal identity-related product. The association between focal and alternative identities was also proved to have a positive effect on the accessibility of alternative identity (*b* = 0.4101, *t* = 9.511, *p* = 0.0000); thus, Hypothesis 1 was also accepted under the moderation effect of alternative discrepancy. Besides, the results showed that the accessibility of alternative identity had a positive effect on the intent to purchase the product concerned with the alternative vs. focal identity (*b* = 0.1547, *t* = 2.6798, *p* = 0.0078 < 0.05); Hypothesis 2 was accepted, this finding suggested individuals were more likely to purchase products that were related to the alternative rather than the focal identity when the alternative identity was accessible for them.

Besides, Hypothesis 4 was also accepted. The alternative identity discrepancy played a moderating role in the effect of alternative identity accessibility on purchasing product concerned with the alternative vs. focal identity (*b* = 0.4248, *t* = 0.1517, *p* = 0.000 < 0.001). More specifically, as shown in Table 5B, the conditional effects for the alternative identity discrepancy was significant at high (+1 SD), mean and low (–1 SD) level of the alternative identity discrepancy [High discrepancy = 0.6647, 95%, CI (0.5284, 0.8009), Mean discrepancy = 0.1547, 95%, CI (0.0411, 0.2683), Low discrepancy = –0.3553, 95%, CI (–0.4818, –0.2287)]; this finding suggested that when the alternative identity discrepancy increased, the effect of accessibility of alternative identity on the alternative identity-related product purchase intent would also increased.

**TABLE 5B:** Results of testing the alternative identity discrepancy’s moderating role.

Moderator: Alternative identity discrepancy						
**Moderator level**	**Effect**	**Se**	***t***	***p***	**LLCI**	**ULCI**

−1.2004 (−1 SD)	−0.3553	0.0643	−5.5275	0.0000	−0.4818	−0.2287
0.000	0.1547	0.0577	2.6798	0.0078	0.0411	0.2683
1.2004 (+1 SD)	0.6647	0.0692	9.6009	0.0000	0.5284	0.8009

Additionally, combining with the results shown in Tables 4A,B, this study also found a different mediation effect of alternative identity in the effect of focal identity on the accessibility of alternative identity. The mediation effect results were shown in Tables 6A,B. The index of conditional moderated mediation for alternative identity clarity was not significant (*b* = 0.0409, 95%, LLCI = –0.0104, ULCI = 0.1015). The index of conditional moderated mediation for alternative identity clarity was significant (*b* = 0.1742, 95%, LLCI = 0.1121, ULCI = 0.2291). More specifically, when the alternative identity discrepancy was significant at high (+1 SD = 1.2004) and low (–1 SD = –1.2004), and not at mean [Low discrepancy = –0.1457, 95%, CI (–0.2388, –0.0679), Mean discrepancy = 0.0634, 95%, CI (–0.0131, 0.1219), High discrepancy = 0.0546, 95%, CI (0.1584, 0.3743)]. That is, with the increase of alternative identity discrepancy, the mediation effect of accessibility of alternative identity was enhanced with the increase of alternative identity discrepancy.

**TABLE 6A:** Results of the mediation effect of the accessibility of alternative identity under alternative identity clarity situation.

	Mediator: Accessibility of alternative identity			
	**Index of moderated mediation**			
	
	**Index**	**Se**	**LLCI**	**ULCI**

AIC	0.0409	0.0283	−0.0104	0.1015

**Moderator level**	**Effect**	**Se**	**LLCI**	**ULCI**

−1.1766 (−1 SD)	0.0037	0.0181	−0.0409	0.0350
0.000	0.0519	0.0327	−0.0161	0.1153
1.1766 (+1 SD)	0.1000	0.0634	−0.0300	0.2237

**TABLE 6B:** Results of the mediation effect of the accessibility of alternative identity under alternative identity discrepancy situation.

	Mediator: Accessibility of alternative identity			
	**Index of moderated mediation**			
	
	**Index**	**Se**	**LLCI**	**ULCI**

AID	0.1742	0.0297	0.1121	0.2291

**Moderator level**	**Effect**	**Se**	**LLCI**	**ULCI**

−1.1766 (−1 SD)	−0.1457	0.0436	−0.2388	−0.0679
0.000	0.0634	0.0342	−0.0131	0.1219
1.1766 (+1 SD)	0.2726	0.0546	0.1584	0.3743

## Research Summary

To examine the moderated mediating effect of our dependent variable, we used model 7 to verify the effect of self-concept clarity of the alternative identity and model 14 to verify the effective self-discrepancy of the alternative identity. The results revealed that all hypotheses were accepted. That is, the association between alternative and focal identities had a positive effect on the accessibility of the alternative identity (H1). This conclusion was opposite to those of the immediate previous research. In addition, accessibility of the alternative identity has a positive effect on the intent to purchase products related to the alternative identity vs. the focal identity (H2). Alternative identity accessibility had a significant positive moderating effect as part of the effect of focal identity on the intent to purchase focal identity vs. alternative identity-related products. More specifically, alternative identity was more likely to be accessible for those who perceive a greater association between the focal and alternative identities. This conclusion revealed differences between individualistic western culture and collectivist Chinese culture.

Our findings also revealed that the self-concept clarity about alternative identity moderated the positive effect of the focal identity on the accessibility of alternative identity (H3). Self-discrepancy related to alternative identity moderated the positive effect of accessibility on the intent to purchase focal identity vs. alternative identity-related products (H4). That is, when individuals had high self-concept clarity about the alternative identity, the alternative identity became more accessible for them. A perceived high level of self-discrepancy related to the alternative identity also gave rise to a strong intent to purchase alternative identity- rather than focal identity-related products.

In addition, the results also revealed that the association between focal and alternative identities had a direct effect on the intent to purchase alternative identity vs. focal identity-related products. This positive effect was evident in tests for both self-concept clarity about the alternative identity and self-discrepancy about the alternative identity (*b* = 0.3533, *t* = 7.0207, *p* = 0.0000 < 0.001), (*b* = 0.3229, *t* = 5.3748, *p* = 0.0000 < 0.001). That is, the perceived association between focal and alternative identities also guides individuals’ intent to purchase alternative identity-related products.

## Theoretical Implications

Identity gives human beings meaning and it is more than any inherent biological characteristics (e.g., gender and age). Identity comes into play when individuals interact with others and the outside world. Although previous western studies have demonstrated the importance of identity (Glynn, 2000; Owens et al., 2010; Leary and Tangney, 2012) and studied its effects on individuals’ psychology and behavior (Abrams and Hogg, 1988; Schouten, 1991; Gini, 1998; Reed et al., 2012), other researchers have also studied identity conflict (Fiol et al., 2009; Horton et al., 2014) and the inhibiting effect of identity activation (Ambady et al., 2001; Forehand et al., 2002). That is, the activation of one identity has an inhibiting effect on other identities. However, this is not always the case. As mentioned in the literature, identity means different things in different cultures and affects individuals’ behavior differently. The previous studies on this topic are limited. Some have verified the impact of individualistic culture, proving that individuals in western countries are more likely to focus on their own goals and less likely to pay attention to contextual factors. Other researches discussed the association of and interactions between focal and alternative identities, the effect of self-concept clarity of alternative identity on consumers’ intent to purchase focal identity vs. alternative identity-related products, and the interaction of alternative identity accessibility and alternative identity self-discrepancy in this process. These studies had diverse outcomes, although they demonstrated that individuals living in countries with Chinese culture are more likely to focus on others rather than themselves.

Identities sometimes overlap, and their similarity leads to an association between them (Saint Clair and Forehand, 2020). In this study, we demonstrate that when identities are associated, alternative identities become accessible when one of them is primed, and the stronger the association is, the more accessible the alternative identity becomes. Our research also verifies the moderating role of self-concept clarity of the alternative identity and the effect of focal identity on alternative identity accessibility. Thus, we extend the literature on identity accessibility, conflict, and clarity.

The previous researchers demonstrated the effect of accessibility of identity, which, like identity salience, promotes positive attitudes toward the identity (Wheeler et al., 2005) and behaviors consistent with this identity rather than other identities (Zhang and Khare, 2009). That is, access to early identity-related information hinders access to later identity-related information (Hoyer and MacInnis, 2008). Few researchers have highlighted the role of alternative identity accessibility. This study demonstrates that the accessibility of an alternative identity diverts individuals’ attention from the focal to the alternative identity, thereby increasing the intent to purchase products related to the alternative identity.

Accessibility of the alternative identity results in relatively lower perceived importance of the focal identity and a lower intent to buy products related to the focal identity. We also verify the moderating role of self-discrepancy of the alternative identity in the effect of alternative identity accessibility on the intent to purchase focal identity vs. alternative identity-related products. Thus, we extend the literature on identity-based consumption, conflict, and discrepancy.

Identity-based consumption is still a common and popular topic in research on consumers’ daily lives, attracting much attention. Thus, identification of factors that affect identity-based consumption and relationships between identities may enrich our understanding of consumer behavior. The findings and conclusions of this research provide a meaningful reference for other scholars in this field.

## Practical Implications

In this study, we discuss and verify the relationships among the association between focal and alternative identities, alternative identity accessibility, self-concept clarity of alternative identity, self-discrepancy of alternative identity, and the intent to purchase focal identity vs. alternative identity-related products. The results differ from those of western studies and we offer particular suggestions and contributions to marketers in countries with Chinese culture.

First, marketers should develop relevant advertising and strategies to activate identities consistent with their products. Better product fit will more easily attract consumers’ attention to target products (Grier and Deshpandé, 2001). Instinctively, when consumers are exposed to products that are consistent with their alternative identities, they shy away from products that are inconsistent with their activated identities (Aaker et al., 2000). However, this study shows that individuals with activated identities are more likely to choose products consistent with their alternative identities when those alternative identities are associated with their focal identities. Based on our findings, marketers in the countries with Chinese culture can develop strategies to manipulate the association of consumers’ identities, creating marketing campaigns to attract their attention to products consistent with their alternative identities. In addition, the repositioning of a single target product to a multi-identity product can effectively avoid the problem of identity positioning.

Second, although self-concept clarity has been considered as a stable individual trait (Campbell et al., 1996), in this study, we examine its moderating role on the relationship between alternative identity and focal identity and the effect of this relationship on alternative identity accessibility. The previous studies argued that more attention to oneself can clarify one’s identity (Emery et al., 2015). Based on our findings, marketers can direct consumers’ attention to the alternative identity through strategies, reducing the impact of the focal identity on targeted products.

Identity discrepancy theory proposed that individuals are motivated to reduce the deficiency between their actual and ideal identities (Moretti and Tory Higgins, 1990; Bessenoff and Snow, 2006). In this study, we find that the perceived deficiency of the alternative identity positively moderates the intent to purchase alternative identity-related products. Thus, we suggest that marketers can arouse consumers’ perceptions, help them focus on alternative identities consistent with targeted products, and guide them to identify deficiencies with their alternative identities.

## Limitations and Directions for Future Research

As with previous research on identity, although this study has implications for identity theory and product management, there still remain limitations in this study.

First, the empirical experiment underlying this focused on the conscious condition. That is, we only study individuals’ conscious intent to purchase products related to the focal identity vs. alternative identity, not their subjective consciousness of various factors. Conscious thought is considered as a reasoning process, which is slower, deliberate, and controlled. Unconscious thought is seen as an intuition process, which is rapid and automatic. It seems that things “happen” to us without our control, and associations related to those things arise in the human mind. Unconscious thoughts tend to lead to certain decisions based on working memory (Evans and Stanovich, 2013). Therefore, conscious and unconscious thoughts lead to different outcomes in terms of goal pursuit. Previous research considered cognitive bias as a cue to these differences. For example, Payne et al. (2008) proposed that different outcomes arise due to how conscious and unconscious thoughts contribute to decision-making. Thus, future research should explore the different effects of focal identity associated with alternative identity on the intent to purchase focal identity vs. alternative identity-related products, making a comparison between the conscious and unconscious conditions.

Second, in this study, we only verify the moderating effects of self-concept clarity of alternative identity and self-discrepancy of alternative identity. The results showed that both of these constructs have positive moderating effects, but the values on the index of conditional moderated mediation for self-concept clarity of alternative identity are not significant. Thus, future researchers may want to explore the simultaneous effects of these two moderators, to determine whether our results for the moderated mediating effect of self-concept clarity of the alternative identity hold under other conditions.

Third, in this study, we only examine the moderating role of self-concept clarity of the alternative identity in the relationship between focal identity and alternative identity accessibility. The moderating role of self-discrepancy of the alternative identity in the relationship between identity accessibility and the intent to purchase products related to focal and alternative identities also deserves further study. Other moderators may also influence the above process.

Due to the various factors, we only focus on Chinese culture, comparing the conclusions and composition of the previous articles based in a western setting when discussing the differences in influence between Chinese and western cultures. However, as our findings are applicable to the field of marketing, the comparison of cultural differences within groups could also be conducted. Finally, this empirical study was conducted based on the following two identities: The worker identity and household head identity. However, the same conclusion may not be reached if other associated identities replaced these two identities.

## Data Availability Statement

The raw data supporting the conclusions of this article will be made available by the authors, without undue reservation.

## Author Contributions

FC, LW, and CY contributed to the conception and design of the study. CY organized the database. XL performed the statistical analysis. FC wrote the first draft of the manuscript. FC, XL, and CY wrote sections of the manuscript. All authors contributed to manuscript revision, read, and approved the submitted version.

## Conflict of Interest

The authors declare that the research was conducted in the absence of any commercial or financial relationships that could be construed as a potential conflict of interest.

## Publisher’s Note

All claims expressed in this article are solely those of the authors and do not necessarily represent those of their affiliated organizations, or those of the publisher, the editors and the reviewers. Any product that may be evaluated in this article, or claim that may be made by its manufacturer, is not guaranteed or endorsed by the publisher.
